# Genotypic and Phenotypic Evaluation of Biofilm Production and Antimicrobial Resistance in *Staphylococcus aureus* Isolated from Milk, North West Province, South Africa

**DOI:** 10.3390/antibiotics9040156

**Published:** 2020-04-02

**Authors:** Marie Ebob Agbortabot Bissong, Collins Njie Ateba

**Affiliations:** 1 Antibiotic Resistance and Phage Biocontrol Research Group (AREPHABREG), Department of Microbiology, School of Biological Sciences, North-West University—Mafikeng Campus, Private Bag X2046, Mmabatho 2735, South Africa; collins.ateba@nwu.ac.za; 2 Department of Biomedical Science, University of Bamenda, Bambili P. O. Box 39, Cameroon; 3 Food Security and Safety Niche Area, Faculty of Natural and Agricultural Sciences, North-West University, Mmabatho, Mafikeng 2735, South Africa

**Keywords:** *Staphylococcus aureus*, biofilm, antimicrobial resistance, genes, milk, public health

## Abstract

**Background**: Biofilm formation in *S. aureus* may reduce the rate of penetration of antibiotics, thereby complicating treatment of infections caused by these bacteria. The aim of this study was to correlate biofilm-forming potentials, antimicrobial resistance, and genes in *S. aureus* isolates. **Methods**: A total of 64 milk samples were analysed, and 77 *S. aureus* were isolated. **Results**: Seventy (90.9%) isolates were biofilm producers. The *ica* biofilm-forming genes were detected among 75.3% of the isolates, with *ica*A being the most prevalent (49, 63.6%). The *ica*B gene was significantly (*P* = 0.027) higher in isolates with strong biofilm formation potentials. High resistance (60%–90%) of the isolates was observed against ceftriaxone, vancomycin, and penicillin, and 25 (32.5%) of *S. aureus* showed multidrug resistance (MDR) to at least three antibiotics. Five resistance genes, namely *blaZ* (29, 37.7%), *vanC* (29, 37.7%), *tetK* (24, 31.2%), tetL (21, 27.3%), and *msrA/B* (16, 20.8%) were detected. Most MDR phenotypes possessed at least one resistance gene alongside the biofilm genes. However, no distinct pattern was identified among the resistance and biofilm phenotypes. **Conclusions**: The high frequency of potentially pathogenic MDR *S. aureus* in milk samples intended for human consumption, demonstrates the public health relevance of this pathogen in the region.

## 1. Introduction

*Staphylococcus aureus* is a Gram-positive bacterium that colonizes the skin, anterior nares, and mucous membranes of a variety of animal species [1,2]. They are facultative pathogenic organisms in both animals and humans and therefore cause a variety of diseases in their hosts [3]. *S. aureus* causes mastitis in dairy cattle, and it is known to be one of the most important etiological agents of intra-mammary infections in ruminants [4]. As a result, *S. aureus* infections present major economic and severe public health challenges to the milk and dairy sectors [4]. Infected mammary glands are the main reservoir for infection; however, the contamination of dairy products can occur anywhere in the food chain, especially during milking procedures [3]. The consumption of contaminated milk may present with serious health hazards to humans [4]. The public health significance of *S. aureus* is amplified by the fact that in humans, this bacterium is associated with both nosocomial and community-acquired infections [5]. 

The potential of *S. aureus* to cause disease is based on its ability to produce a wide variety of virulence factors that contribute to bacterial invasion [6]. These include factors that are responsible for the attachment, adherence, and biofilm formation [6]. Biofilm formation has been reported to enhance the virulence of bacterial species, including *S. aureus* [6,7]. Biofilm formation in *S. aureus* involves three major stages: attachment, maturation, and dispersion of bacterial cells [7]. The fundamental step in *S. aureus* biofilm formation is initial attachment, which is accomplished by the expression of different Microbial Surface Components Recognizing Adhesive Matrix Molecules (MSCRAMMs) [7]. *S. aureus* initially adheres to each other and then widen to structurally dynamic biofilm structures during the later phases of adherence. The maturation of the biofilm matrix into multi-layered patterns is initiated by the polysaccharide intercellular adhesin (PIA), synthesized from *β*-1, 6- linked N-acetyld-glucosamines (PNAG) [8]. The synthesis of PIA is mediated by the intercellular adhesin (ica) locus, which comprises four core genes, namely *icaA*, *icaD*, *icaB*, and *icaC*, as well as a regulatory gene, (*icaR*) [9]. These genes encode the corresponding proteins ICAA, ICAD, ICAB, and ICAC. The production of slime is facilitated by the coexpression of *icaA* and *icaD* genes [10]. The role of icaB is not fully elucidated, but icaC acts as a receptor for polysaccharides [9]. It has been shown that strains harbouring the icaADBC cluster are potential biofilm producers [10]. In addition, the biofilm-associated protein (*Bap*) is vital for the primary attachment of *S. aureus* and biofilm formation [11,12].

Antimicrobial resistance in *S. aureus* is a major veterinary, as well as public, health concern worldwide since multi-resistant strains present a severe challenge to effective treatment [13,14,15]. Antibiotics are extensively used in food-animal production, and this has resulted in the rise of antibiotic-resistant *S. aureus* in food animals and foods of animal origin [16]. Antimicrobial resistance in methicillin resistant *S. aureus* (MRSA) strains is due to the production of penicillin-binding protein 2a (PBP2a), which is encoded by the *mecA* gene located on the mobile element of the staphylococcal chromosome cassette mec (*SCCmec*) [17]. The presence of these genes in MRSA make them resistant to multiple antibiotics, particularly all ß-lactam antibiotics. It has been suggested that cattle may serve as a source of MDR and new MRSA strains in humans [18]. Therefore, colonization of dairy cattle with antimicrobial-resistant *S. aureus* impacts milk production and may additionally represent an infection hazard to people who work in close contact with cows or consume raw milk [18]. 

The colonization of dairy cattle and subsequent contamination of raw milk by pathogenic *S. aureus* remains an important issue for both the dairy producers and the public health sector [19]. The formation of biofilm reduces the rate of penetration of antibiotics, thereby complicating treatment of infections caused by these bacteria [6,19,20]. Colonization impacts negatively on milk production and may additionally represent an infection hazard to people who work in close contact with cows or consume raw milk [18]. The food-animal production industry in South Africa is fast growing in order to meet the demand of meat supply to the increasing population [13,16]. Most of the dairy farms located in the Mafikeng area are small-scale farms owned by independent farmers who usually supply fresh milk to processing plants, as well as to the local communities for direct consumption. In such cases, if proper hygienic practices are not observed during the milking process, milk intended for human consumption may become contaminated and eventually pose serious health problems to the consumers. This highlights the importance of the screening of milk and other animal products, for the presence of pathogenic biofilm producing *S. aureus*.

In South Africa, *S. aureus* has been detected from varied sources, including bovine milk [21,22,23], pigs [24], beach water [25], and human samples [23,26,27]. However, studies reporting the biofilm-producing capabilities of *S. aureus* in Southern Africa are limited [27]. Naicker et al reported strong biofilm production among a large proportion (57%) of clinically invasive *S. aureus* isolates belonging to the spa clonal complex 064 [27]. The present study is aimed at expanding on previous investigations in the area, by determining the genotypic and phenotypic characteristics of biofilm producing and antimicrobial-resistant *S. aureus* isolated from milk. 

## 2. Results

### 2.1. Isolation of S. aureus 

*S. aureus* was isolated from all the unpasteurized milk samples, and only 7 out of 24 (29.2%) pasteurized milk samples showed growth. A total of 77 *S. aureus* were isolated from the milk samples, out of which 7 (9.1%) and 70 (90.9%) isolates were from pasteurized and unpasteurized milk, respectively (*P* < 0.05). The distribution of isolates with respect to the study location presented in Table 1 revealed that more of the isolates (33, 42.9%) were from Mafikeng, while only two isolates (2.5%) were from Zeerust.

### 2.2. Phenotypic Biofilm Production

Out of 77 *S. aureus* isolates tested for phenotypic biofilm formation, 70 (90.9%) were biofilm producers, while seven (9.1%) were non-biofilm producers. The biofilm producers were further classified as “Strong” (66, 94.3%), “Moderate” (4, 5.7%), and “Weak” (0, 0.0%) producers. The distribution of biofilm formation in *S. aureus* strains isolated from the different study location did not show any significant difference (*P* = 0.526).

### 2.3. Detection of Biofilm Genes

**Figure 1:**
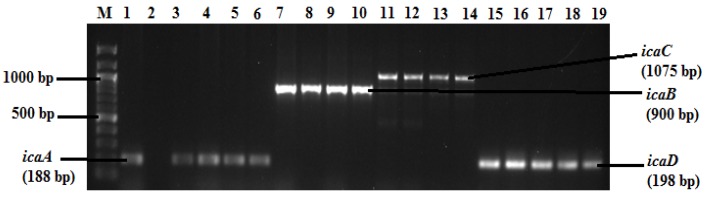
Agarose (1% w/v) gel of the *ica* gene products of *S. aureus* isolates. Lane M = DNA marker (100 base pairs DNA Ladder); Lane 1 = *icaA* amplicon of positive control (*S. aureus* ATCC 25923); Lane 2 = *icaA* amplicon of negative control (*E. coli* ATCC 25922); Lanes 3–6 = *icaA* amplicons; Lane 10 = *icaB* amplicons; Lanes 11–14 = *icaC* amplicons; Lanes 15–19 = *icaD* amplicons.

Five biofilm genes were detected among *S. aureus* isolates, and the electrophoretic patterns of the *ica* genes are presented in Figure 1. The *ica* genes were detected in 58 (75.3%) of the isolates, among which *icaA* was the most prevalent (49, 63.6%), followed by *icaB* (48, 62.3%), *icaD* (43, 55.8%) and *icaC* (30, 38.9%). On the other hand, the *bap* gene showed the lowest rate of detection (12, 15.6%). 

### 2.4. Cluster Analysis of Biofilm Genes in S. aureus Isolated from Milk

**Figure 2:**
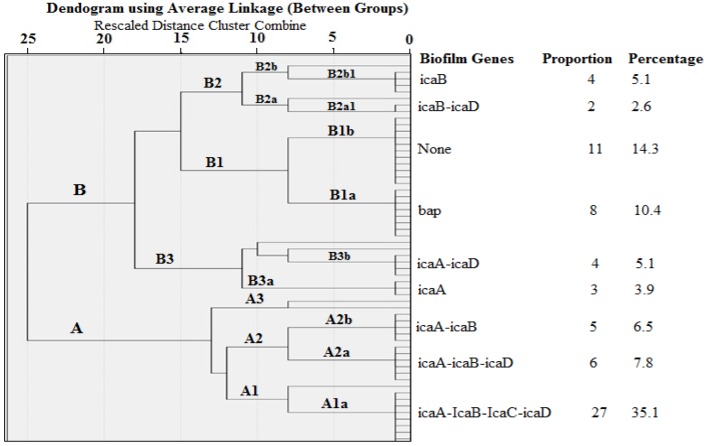
A dendrogram showing the clustering of biofilm genes based on OD_680_ of *S. aureus* isolates (the dendrogram was obtained by using the unweighted paired group method).

Several genotypes with various biofilm gene combinations were observed in a hierarchical cluster analysis, as presented in Figure 2. However, the most prevalent (27, 31.5%) was “Genotype A1a”, which comprises all the *ica* genes (icaA, icaB, *icaC*, and *icaD*). Interestingly, the *bap* gene occurred independently of the *ica* genes in 8 out of 12 (66.7%) *S. aureus* isolates. Furthermore, 11 out of 77 (14.3%) *S. aureus* isolates did not present with any of the biofilm genes. Only 4 out of 77 (5.1%) *S. aureus* isolates possessed both *icaA* and *icaD* genes (Genotype B3b). 

### 2.5. Relationship between Biofilm Formation and Biofilm Genes in S. aureus Isolates

The distribution of various biofilm genes with respect to biofilm formation in *S. aureus* isolates is presented in Table 2. Although there was no significant difference (*P* > 0.05) in the occurrence of the iacA, *icaC*, iacD, and *bap* among various categories of biofilm-producing *S. aureus*, these genes were generally more common in biofilm-producing isolates than in non-biofilm-producing isolates. On the other hand, the frequency of *icaB* was significantly higher in “strong biofilm producers” (44, 69.7%) than in “Moderate” (2, 50.0%) and “None” producers (0, 0.0%).

### 2.6. Antibiotic Susceptibility Test (AST)

**Figure 3:**
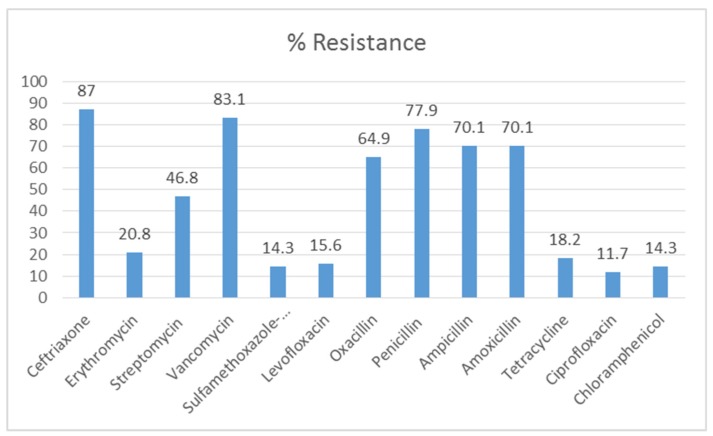
Antimicrobial resistance of *S. aureus* isolates.

All 77 *S. aureus* isolates were resistant to at least one of the 13 antibiotics. High resistance (60%–90%) was observed against ceftriaxone (87%), followed by vancomycin (83.1%) and penicillin G (77.9%) (Figure 3). Other members of the class penicillin (amoxicillin, ampicillin, and oxacillin) showed similar resistance patterns to penicillin G. In contrast, low resistance was observed against ciprofloxacin (11.7%), chloramphenicol (14.3%), and sulfamethoxazole–trimethoprim (14.3%). Meanwhile, twenty-five (32.5%) of the *S. aureus* isolates showed multidrug resistance (MDR) to at least three antibiotics of different classes. 

A total of 30 were selected to be tested for inducible clindamycin resistance (D test). None of the isolates showed inducible clindamycin resistance. However, three phenotypes were observed: Phenotype “R” (9, 30%) which were resistant to both erythromycin and clindamycin and are indicative of constitutive clindamycin resistance; Phenotype “I” (8, 26.7%), which showed intermediate resistance to clindamycin but were susceptible to erythromycin; and Phenotype “S” (13, 43.3%), which were sensitive to both erythromycin and clindamycin. 

Five resistance genes were detected in the isolates, with the following frequencies: *blaZ* (29, 37.7%), *vanB* (29, 37.7%), *tetK* (24, 31.2%), *tetL* (21, 27.3%), and *msrA/B* (16, 20.8%). On the other hand, *vanA* and *mefA* genes were not detected among the isolates. The distribution of the resistance genes with respect to phenotypic resistance to the corresponding antibiotic was not statistically significant (*P* > 0.05).

### 2.7. Relationship between Penicillin Diameter, blaZ Genes, and Beta Lactamase Production

**Figure 4:**
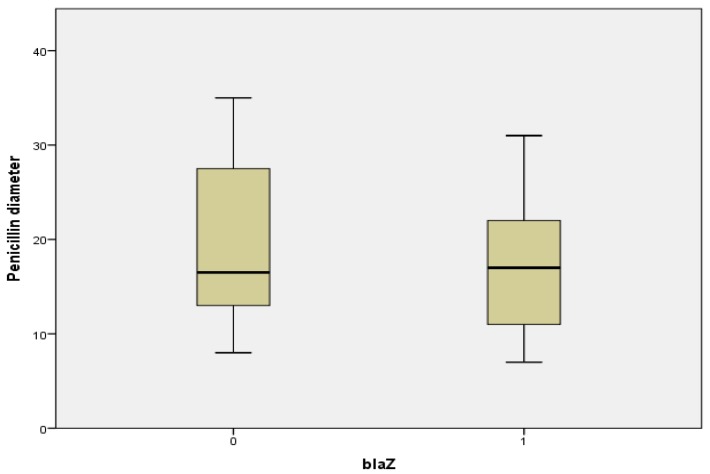
A Boxplot of penicillin diameter and *blaZ* gene (0=*blaZ*-negative, 1=*blaZ*-positive).

Figure 4 shows the Boxplot of penicillin diameter (PD) of *blaZ*-negative and *blaZ*-positive *S. aureus* isolates. Although the median PDs of *blaZ*-negative and *blaZ*-positive isolates are slightly similar, the interquartile range of *blaZ*-negative isolates is higher than that of *blaZ*-positive isolates. In addition, the Mann–Whitney U Test revealed that the PD of blaZ-negative isolates (Mean Rank = 40.34) was higher than that of blaZ-positive isolates (Mean Rank = 36.78), though this difference was not significant (*U* = 631.5, *P* = 0.497). 

A total of 56 (72.7%) of the isolates were positive for beta lactamase production (zone-edge test). There was a relationship between *blaZ* and beta lactamase production (χ^2^ = 4.262, *p* = 0.039). More (25/29, 86.2%) of the isolates that are positive for the *blaZ* gene also expressed beta lactamase production (Table 3). 

### 2.8. Profiling of Biofilm Formation and Resistance Patterns of MDR S. aureus Isolates

The resistance and biofilm profiles of the 25 MDR isolates were assessed, and it was observed that the majority (11/, 44%) belong to the cef-van-oxa phenotype, which showed multidrug resistance to ceftriaxone, vancomycin, and oxacillin (including other penicillins) (Table 4). Most of the MDR phenotypes also possessed at least one of the resistance genes, alongside the biofilm genes. Although no distinct pattern was identified among the resistance and biofilm phenotypes, most strains that showed moderate or no biofilm production did not have the *ica* genes (Table 4).

## 3. Discussion

*S. aureus* is an important cause of diseases in both animals and humans [2,3,6,20]. The colonization of dairy cattle and subsequent contamination of raw milk by pathogenic *S. aureus* remains an important issue for both the dairy producer and public health [19]. The potential of *S. aureus* to cause disease is based on its ability to produce a wide variety of virulence factors that contribute to the bacterial invasion [6,19]. Biofilm formation has been reported to enhance the virulence of *S. aureus*, and strains that form biofilms are more resistant to antibiotics, disinfectants, and other environmental factors [15]. The *ica* genes play a major role in slime production in *S. aureus*. It is known that strains harbouring the icaADBC cluster are potential biofilm producers [10]. In addition, the biofilm-associated protein (*bap*) is vital for the primary attachment of *S. aureus* and biofilm formation [11,12]. 

In the present study, the genotypic and phenotypic biofilm-producing characteristics of *S. aureus* isolated from raw unpasteurized and pasteurized milk samples collected from farms and shops in four major localities in the North West Province of South Africa were evaluated. *S. aureus* was isolated from all (32, 80%) unpasteurized milk samples, while only 7 out of 24 (29.2%) pasteurized milk samples showed growth. It is common to observe higher rates of isolation in unpasteurized than in pasteurized milk due to the effect of heat in reducing the microbial load of pasteurized milk. Similar observations have been made in previous studies [14,21]. In a related study, Akindolire and colleagues reported 75% isolation rate of *S. aureus* from raw milk samples, 29% from bulk milk samples, and 13% from pasteurized milk [21]. However, a lower prevalence (22.0%) of *S. aureus* in raw milk collected from healthy cows has been reported [28]. It is worth noting that the isolation of *S. aureus* from 80% of the milk samples from lactating cows in four prominent farms in the North West Province and in 29.2% of processed milk meant for human consumption demonstrates the relevance of this pathogen in the region. Besides, more of the isolates (33, 42.9%) were from samples from Mafikeng. Consequently, further research is necessary to explore methods of controlling *S. aureus* in raw milk, as well as dairy products, in the region.

Phenotypic biofilm production in *S. aureus* has been extensively studied by using the Congo Red plate assay, which is reported to be highly subjective [29,30,31], and as a result, microtitre plate assay (MPA) is considerably used as the gold standard for phenotypic biofilm analyses. In the current study, biofilm production was assessed by using the MPA, and it was observed that about 90% of *S. aureus* isolates produced biofilms, out of which the majority (66/70, 94.3%) were classified as “Strong biofilm producers”. Similar findings have been previously reported in which most *S. aureus* strains from milk were biofilm producers [31,32,33,34]. In one of such studies, all 20 *S. aureus* strains were biofilm producers and 11 out of 20 (55%) were classified as strong biofilm producers [33]. In another recent study in Egypt, 69.8% of *S. aureus* from clinical specimens were biofilm producers; however, contrary to our findings, most (16/43, 37.2%) of their isolates demonstrated weak biofilm production [15]. The discrepancies in the categorization of the biofilm phenotypes could result from differences in the interpretation of results. As such, the standardization of the methods and interpretation of biofilm formation is crucial. 

In this study, *ica* genes (icaA, icaB, *icaC*, and *icaD*) were detected among 75.3% of *S. aureus* isolates, among which *icaA* was the most prevalent (49, 63.6%). Our findings are similar to other reports in which the *ica* genes were detected in all isolates [33,34]. While Gowishankar and colleagues detected the *ica* genes in 84.13% of *S. aureus* isolates in India [35], Avila-Novoa detected the genes in 52.3% of isolates in Brazil [29]. However, the proportion (4, 5.1%) of isolates possessing the *icaA* and *icaD* genes associated with slime formation was relatively low in this study, as compared with some previous studies [31,33,34]. These results suggest that strains of *S. aureus* may present with different capacities to form biofilms based on their source (human or animals) and geographical origin. Further studies, including varied sources, are needed to fully clarify this assertion. 

In line with other studies [31,33], results from this study showed that the incidence of the *bap* gene was low (12, 15.6%). This might indicate that the *ica*-dependent mechanism may be primarily responsible for the adhesion and biofilm formation in these isolates, although not all genes responsible for biofilm formation were tested. Despite the pro-biofilm production role of the *ica* gene, coupled with the fact that other possible biofilm-producing genes may be harboured by stains displaying moderate or no biofilm traits, it is recommended that these isolates should be screened for the presence of the *mecA*, *femA*, *gyrA*, *gyrB*, *grlA*, *cfr, ermA*, *ermB*, and *ermC* genes. However, contrary to our reports, the study by Li et al. reported that the *bap* gene was detected in 43.9% of biofilm-positive *S. aureus* strains, confirming the importance of the *bap* gene in biofilm formation [36].

Interestingly, our cluster analysis revealed that the predominant genotype (A1a) consists of strains that possess all the four *ica* genes. This confirms the importance of the *ica* genes in biofilm formation. In addition, the majority of the isolates that possessed the *bap* genes did not have the *ica* genes; suggestive of an *ica*–*bap* gene independent mechanism. Although none of the biofilm genes tested were detected in some (14.3%) of the isolates, the expression of biofilm formation in these isolates could be mediated by other genes. Generally, the biofilm genes were more common in biofilm-producing isolates than in non-biofilm-producing isolates. These results are concurrent with previous reports [34,36]. The screening of milk for the detection of biofilm-related genes and biofilm-producing *Staphylococcus* species is important because biofilm facilitates the adhesion to glandular breast tissue and biomaterials, thereby enhancing the bacterial virulence [6,20]. In addition, the presence of biofilms confers resistance of the bacterial community to antibiotics, hence complicating treatment [19].

The misuse and or overuse of antibiotics in livestock is known to have contributed tremendously to the emergence of resistance strains. Antibiotic usage in food-producing animals tend to be increasing worldwide, and in South Africa, two-thirds of antibiotics are administered through animal feed [13]. Previous data have shown that even the antibiotics which are banned in other countries, such as growth promoters, were still being used in South Africa [37]. Consequently, the screening of animal products for antimicrobial resistant pathogens is pertinent. In this study, thirteen antibiotics belonging to nine classes of antimicrobials were screened against *S. aureus* isolated from raw and pasteurized cattle milk, and resistance was observed against all the antibiotics. However, the highest resistance was against ceftriaxone, vancomycin, and penicillin G. High resistance was also observed against other penicillins, such as amoxicillin, ampicillin, ampicillin, and oxacillin. Some of these antibiotics are registered in South Africa as stock remedies—readily available for use by the lay public [16,37]. This could explain the high resistance observed against these antibiotics in our study. Although cephalosporins are rarely used in poultry, the high resistance to ceftriaxone observed in this study may be due to the transfer of a multidrug-resistant plasmid, or to extended-spectrum beta-lactamase (ESBL) production. In this study, vancomycin showed a high resistance of 83.1%. The mechanism of resistance to glycopeptides is not well defined, but it is suggested that resistance could be through the acquisition of the mobile genetic elements of vancomycin-resistant *Enterococcus* that cause changes in the metabolism and cell wall of *Staphylococcus* [38]. These results are in line with reports from previous studies on animal and clinical samples [22,33,39,40]. Ateba and colleagues recorded a moderate to high percentage (39%–100%) of resistance to methicillin, ampicillin, penicillin G, sulfamethoxazole, oxytetracycline, and erythromycin among *S. aureus* isolates from communal farms [22]. 

Five resistance genes, *blaZ*, *vanB*, *tetK*, *tetL*, and *msrA*, were equally detected in our isolates, in relatively high proportions. However, the distribution of these genes with respect to phenotypic antibiotic resistance was not statistically significant. This is contrary to studies by Zehra et al. which reported a statistically significant relationship between phenotypes and genotype resistance pattern in *S. aureus* strains [40]. The phenotypic resistance in strains without the genes may be caused by other attributes, such as point mutations, biofilm formation, or antibiotic tolerance. The *vanA* and *mefA* genes were not detected in this study, and this is similar to previous reports [41].

In this study, the diameter of the zone of inhibition of penicillin (PD) was higher in *S. aureus* isolates without the *blaZ* gene than those with the *blaZ* gene. In a similar study, Ferreira et al. reported that *S. aureus* isolates with a penicillin diameter less than 28 mm carried the *blaZ* gene [42]. However, a large inhibition halo did not rule out the presence of the *bla*Z gene, as reported by other colleagues [43,44]. These reports suggested that changing the breakpoint of the penicillin inhibition halo from 29 to 35 mm would improve sensitivity of the test. Moreover, our results revealed a relationship between *blaZ* and beta lactamase production, with more (25/29, 86.2%) of the isolates positive for the *blaZ* gene also expressing beta lactamase production. Meanwhile, 56 (72.7%) of the isolates were positive for beta lactamase production by the zone-edge test. Similar results have been reported in which Marques et al. recorded a 100% positive zone-edge test among *S. aureus* isolates from bovine mastitis [33]. 

## 4. Materials and Methods 

### 4.1. Study Site

This study was conducted in the North-West University, Mafikeng, South Africa. Milk samples were collected from dairy farms and shops in Lichtenburg, Mafikeng, and Zeerust, which are all located in the Northwest Province of South Africa. Laboratory analyses were performed in the Molecular Microbiology Laboratory of the Department of Biological Sciences. 

### 4.2. Sample Collection

Unpasteurized milk samples were collected directly from dairy cattle, as well as from storage tanks in various farms, while pasteurized milk was bought from shops. The samples were transported on ice to the laboratory, for analysis. A total of 64 milk samples were obtained, and out of these samples, 40 were unpasteurized, while 24 were pasteurized.

### 4.3. Isolation and Identification of Staphylococcus aureus Isolates

A ten-fold serial dilutions of each milk sample was performed, using sterile 2% (w/v) peptone water (Biolab, South Africa). An aliquot of 100 µL of the diluted milk was spread-plated on mannitol salt agar (MSA) (Merck, Darmstadt, Germany), and the plates were incubated aerobically at 37 °C for 24 h. Isolates that presented with yellow colonies were subcultured on MSA, and the plates were incubated aerobically at 37 °C for 24 h. Pure colonies were identified by using standard biochemical tests (cellular morphology, catalase test, coagulase test, and mannitol fermentation). All catalase positive, coagulase positive, and Gram-positive cocci in clusters that fermented mannitol were stored in Tryptic soy broth with 40% (v/v) glycerol, at −80 °C, for further analyses. DNA was extracted from the isolates, using the Zymo Research Genomic DNA^TM^ Tissue MiniPrep Kit (Inqaba Biotec, Pretoria, South Africa), and confirmation of *S. aureus* isolates was done by PCR, using the *nuc* gene primers (Table 1). The reactions were prepared in 25 μL volumes made up of 12.5 μL of One Taq^R^ Quickload 2X Master Mix with standard buffer (Biolabs, New England, UK), 0.25 μL of each primer, 1 μL of template DNA, and 11 μL nuclease-free sterile water. Amplifications were performed by using the model- Bio-Rad C1000 Touch^TM^ Thermal Cycler (Bio-Rad Laboratories, Inc., Hercules, California, USA). PCR conditions comprised an initial denaturation at 94 °C for 5 min, followed by 35 cycles of denaturation at 94 °C for 30 s, annealing at 55 °C for 30 s, and extension at 72 °C for 1 min. A final extension step was performed at 72 °C for 10 min. PCR products were stored at 4 °C, until electrophoresis. All PCR reagents were Fermentas products (Thermo Fisher Scientific Inc., Waltham, MA, USA) obtained from Inqaba Biotec Ltd., South Africa.

### 4.4. PCR Detection of Biofilm and Antimicrobial Resistance Genes

All isolates confirmed to be *S. aureus* by the presence of the *nuc* gene were screened for the presence of biofilm genes (*icaA*, *icaB*, *icaC*, *icaD*, and *bap*) and resistance genes (*blaZ*, *vanB*, *tetK*, *tetL*, and *msrA/B*); primer sequences are presented in Table 5. The reactions were prepared in 25 μL volumes made up of 12.5 μL of One Taq^R^ Quickload 2X Master Mix with standard buffer (Biolabs, New England, UK), 1 μL of template DNA, and a final primer concentration of 0.2 μM. Amplifications were performed by using the model Bio-Rad C1000 Touch^TM^ Thermal Cycler (Bio-Rad Laboratories, Inc., Hercules, California, USA). The PCR conditions were the same as those for the *nuc* gene, except for *blaZ* and *tetL* genes, which annealed at 50 °C. All PCR products were resolved by electrophoresis on a 1% (w/v) agarose gel, at 80 volts, for 3 h, using 1X TAE buffer (40 mM Tris, 1 mM EDTA, and 20 mM glacial acetic acid, pH 8.0). A 100 bp DNA molecular weight marker (Thermo Fisher Scientific Inc., Waltham, MA, USA) was included in each gel. The gels were stained in 0.001 µg/mL of ethidium bromide, for 15 min, and the amplicons were visualized, using a Bio-RAD ChemiDoc^TM^ MP Imaging System (Bio-Rad Laboratories Ltd., Watford, UK). 

### 4.5. Phenotypic Biofilm Production

Formation of biofilm by *S. aureus* was evaluated by using a modified version of the microtitre plate (MTP) test described by Christensen et al. [52]. Overnight cultures of the bacterial isolates were diluted 1:1 in Tryptic soy broth. Each well (of a 96-well plate) was filled with 200 uL of the diluted culture. The plates were incubated for 24 h at 37 °C, after which the wells were washed three times with 200 uL of phosphate buffer saline (PBS), pH 7.2. The plates were dried at room temperature for 1 h. The wells were stained by adding 200 uL of 1% crystal violet for one minute, after which excess dye was removed and the wells were rinsed three times with PBS. The optical density was read, using the MB-580 Microtitre plate reader (Heales Technology, Shenzhen, China), at a wavelength of 680 nm. Interpretation of results was done as previously described [36], and results were recorded as none, weak, moderate, and strong biofilm-producing. 

### 4.6. Antibiotic Susceptibility Testing (AST)

All *S. aureus* isolates were screened for susceptibility to the following antimicrobial agents: oxacillin (1 µg), erythromycin (15 µg), vancomycin (30 µg), streptomycin (10 µg), tetracycline (30 µg), levofloxacin (5 µg), sulfamethoxazole-trimethoprim (25 µg), ciprofloxacin (5 µg), penicillin (10 Units), chloramphenicol (30 µg), amoxicillin (10 µg), ceftriaxone (30 µg), and ampicillin (10 µg), by the disk diffusion technique. Suspensions of overnight cultures were prepared and diluted, using sterile normal saline, to obtain the inoculum that was compared with a 0.5 McFarland’s standard. The inoculum was plated on Mueller Hinton agar (Merck, Darmstadt, Germany) and incubated at 37 °C for 18–24 h. The zones of inhibition were measured, and the results were interpreted based on recommended standards [53,54]. 

### 4.7. Phenotypic Evaluation of Beta-Lactamase Production and Inducible Clindamycin Resistance 

The zone-edge test was used to evaluate beta-lactamases production by isolates. Procedures were carried out as previously described [42]. Bacterial inoculum was plated on MHA, and penicillin-G disks (10 Units) were placed at the centre of each plate, and the plates were incubated as in AST described above. The zones of inhibition were observed and results were recorded as follows: Positive—zones with sharp edge; Negative—zones with fuzzy edge.

The Double diffusion (D-test) was used to detect isolates with inducible clindamycin resistance, and procedures were done as described [55]. Bacterial inoculum was plated as in the disk diffusion method. Erythromycin (15 µg) and Clindamycin (2 µg) disks were placed edge-to-edge on the agar plate, at a distance of about 15 mm. The plates were incubated overnight; after that, they were observed for a flat zone of inhibition. 

### 4.8. Statistical Analysis

Data was analysed by using the SPSS version 20 (SPSS, Chicago, USA) statistical package. Descriptive statistic was done for all variables, and the Chi square test was used to determine significant differences between categorical variables, while the Mann–Whitney U test was used to compare means at a 95% confidence level. 

## 5. Conclusions

The *ica* and *bap* genes were detected in *S. aureus* isolated from milk. The presence of these genes in biofilm-producing isolates indicates their importance in biofilm formation in *S. aureus*. High resistance against ceftriaxone, vancomycin, and penicillin, as well as five different resistance genes, were detected among these isolates. The presence of potential biofilm-producing and antibiotic-resistant *S. aureus* in milk intended for human consumption may present with severe health challenges. Consequently, there is a need to enhance control measures, especially in the dairy sector, to curb the spread of pathogenic *S. aureus* and to limit the use of antibiotics.

## Figures and Tables

**Table 1:** Distribution of biofilm formation with respect to study location.

Proportion of Isolates in the Different Biofilm Forming Categories		Location				Total	*P*-Value
		Mafikeng	Rooidepand	Rooigrond	Zeerust		
**Biofilm Formation**	Moderate	3	1	0	0	4	
	None	3	1	2	1	7	0.526
	Strong	27	13	25	1	66	
**Total**		33 (42.9)	15 (19.5)	27 (35.1)	2 (2.5)	77	

**Table 2:** Relationship between biofilm formation and biofilm genes in *S. aureus* isolates.

Biofilm Formation	Number (%) of Isolates Having the Biofilm Gene				
	*icaA*	*icaB*	*icaC*	*icaD*	*bap*
None (*n* = 7)	4 (57.1)	0 (0.0)	0 (0.0)	3 (42.9)	1 (14.3)
Moderate (*n* = 4)	3 (75.0)	2 (50)	3 (75.0)	4 (100)	1 (25)
Strong (*n* = 66)	42 (63.6)	46 (69.7)	27 (40.9)	36 (54.5)	10 (15.1)
Total (*n* = 77)	49 (63.6)	48 (62.3)	30 (38.9)	43 (55.8)	12 (15.6)
*P*-value	0.777	0.027	0.259	0.674	0.084

**Table 3:** Correlation between *blaZ* genes and beta lactamase production.

Phenotypic Characteristic		*blaZ* Gene (% *)		Total
		Negative	Positive	
Beta Lactamase Production	Negative	17 (35.4)	4 (13.8)	21
	Positive	31 (64.6)	25 (86.2)	56
Total		48	29	77

* Percentages calculated across columns.

**Table 4:** Antimicrobial resistance and biofilm profile of MDR *S. aureus* isolates.

MDR Isolate	Resistance Phenotype	Biofilm Production	Resistance Genes					Biofilm Genes				
			*bla* *Z*	*van* *C*	*tet* *K*	*tet* *L*	*Msr* *A/B*	*Ica* Genes				*bap*
								*A*	*B*	*C*	*D*	
Sum 1	cef-str-van-oxa	strong	+	-	-	-	-	+	+	+	+	-
Ctrl 2	cef-str-van-oxa	strong	+	+	+	-	-	+	+	+	+	-
XWL2	cef-str-van-oxa	strong	+	+	+	+	-	+	+	+	+	-
RPN4L1	cef-van-oxa	strong	+	-	-	-	-	-	-	-	-	+
RPN4L2	cef-van-oxa	strong	+	+	-	-	-	+	+	+	-	-
RPN21	cef-oxa-cip-chl	none	-	+	+	+	-	-	-	-	-	+
RPN3	str-van-oxa-tet	strong	+	+	+	+	-	+	+	+	+	-
SUM 2	cef-str-van-oxa	strong	+	-	+	-	-	+	+	+	+	-
RPN2L	cef-van-oxa-tet-chl	strong	-	-	-	-	-	+	+	+	+	-
RPN1L	cef-van-oxa	strong	+	-	-	-	-	+	-	-	-	-
RPN30	cef-ery-str-van-lev-oxa-tet	strong	+	+	+	+	+	-	+	-	-	-
Fa1L	cef-ery-van-oxa-tet	moderate	-	-	+	+	-	-	-	-	-	+
Fa2S	cef-str-van-sul-lev-oxa-cip-chl	strong	-	-	-	-	-	-	+	-	-	-
Bb1LY2	cef-van-oxa	strong	-	-	-	-	-	-	-	-	-	-
LIC3M	cef-van-oxa-cip-chl	strong	+	+	-	-	-	+	+	-	+	-
XY	cef-van-oxa	strong	+	+	+	+	-	-	-	-	-	+
LIC 1	str-van-oxa-tet	strong	-	+	+	+	-	-	-	-	-	+
LIC 2M	cef-str-van	strong	-	-	-	-	-	+	+	-	+	-
KE18	cef-oxa-tet	strong	-	-	+	+	+	-	-	-	-	-
K5	cef-van-oxa	strong	+	+	+	+	-	+	+	-	+	-
K9	cef-van-oxa	strong	-	-	-	-	+	+	+	-	+	-
K10	cef-van-oxa	strong	+	-	-	-	-	+	+	-	+	-
K11	cef-van-oxa	strong	+	+	+	+	-	-	+	-	+	-
K85	cef-van-oxa	strong	+	+	-	-	-	+	+	+	+	-
K87	cef-van-oxa	moderate	+	+	-	-	+	+	-	-	+	-

**Table 5:** Putative virulence and resistance genes and primer sequences used in this study.

Gene	Primer Sequence	Amplicon Size (bp)	Reference
*nuc*	F-GCGATTGATGGTGGATACGGT R-AGCCAAGCCTTGACGAACTAAAGC	279	[45]
*icaA*	F-ACACTTGCTGGCGCAGTCAA R-TCTGGAACCAACATCCAACA	188	[46]
*icaB*	F-AGAATCGTGAAGTATAGAAAATT R-TCTAATCTTTTTCATGGAATCCGT	900	[46]
*icaC*	F-ATGGGACGGATTCCATGAAAAAGA R-TAATAAGCATTAATGTTCAATT	1075	[46]
*icaD*	F-ATGGTCAAGCCCAGACAGAG R-AGTATTTTCAATGTTTAAAGCAA	198	[46]
*bap*	F-CCCTATATCGAAGGTGTAGAATTGCAC R-GCTGTTGAAGTTAATACTGTACCTGC	971	[11]
*blaZ*	F-CAAAGATGATATAGTTGCTTATTCTCC R-TGCTTGACCACTTTTATCAGC	421	[43]
*tetK*	F-GTAGCGACAATAGGTAATAGT R-GTAGTGACAATAAACCTCCTA	360	[47]
*tetL*	F-GTCGTTGCGCGCTATATTCC R-GTGAACGGTAGCCCACCTAA	696	[48]
*mefA*	F-AGTATCATTAATCACTAGTGC R-TTCTTCTGGTACAAAAGTGG	367	[49]
*msrA*	F-CGATGAAGGAGGATTAAAATG R-CATGAATAGATTGTCCTGTTAATT	1733	[49]
*vanA*	F-ATGAATAGAATAAAAGTTGC R-TCACCCCTTTAACGCTAATA	1032	[50]
*vanB*	F-GTGACAAACCGGAGGCGAGGA R-CCGCCATCCTCCTGCAAAAAA	430	[51]
